# Impact of a multidisciplinary management team on clinical outcome in ICU patients affected by Gram-negative bloodstream infections: a pre-post quasi-experimental study

**DOI:** 10.1186/s13613-024-01271-9

**Published:** 2024-03-06

**Authors:** Matteo Rinaldi, Milo Gatti, Tommaso Tonetti, Domenico Nocera, Simone Ambretti, Andrea Berlingeri, Giacomo Nigrisoli, Elisabetta Pierucci, Antonio Siniscalchi, Federico Pea, Pierluigi Viale, Maddalena Giannella

**Affiliations:** 1 Department of Medical and Surgical Sciences, Alma Mater Studiorum University of Bologna, Via Massarenti, 9, Bologna, 40138 Italy; 2 Infectious Diseases Unit, Department for Integrated Infectious Risk Management, IRCCS Azienda Ospedaliero-Universitaria di Bologna, Bologna, Italy; 3 Clinical Pharmacology Unit, IRCCS Azienda Ospedaliero-Universitaria di Bologna, Bologna, Italy; 4 Anesthesiology and General Intensive Care Unit, IRCCS Azienda Ospedaliero-Universitaria di Bologna, Bologna, Italy; 5 Microbiology Unit, IRCCS Azienda Ospedaliero-Universitaria di Bologna, Bologna, 40138 Italy; 6 Anesthesia and Intensive Care Medicine, IRCCS Azienda Ospedaliero-Universitaria di Bologna, Bologna, 40138 Italy

**Keywords:** Multidisciplinary management team, Critically ill patients, Gram-negative bacteraemia

## Abstract

**Background:**

Bloodstream infections (BSIs) by Gram-negative pathogens play a major role in intensive care patients, both in terms of prevalence and severity, especially if multi-drug resistant pathogens are involved. Early appropriate antibiotic therapy is therefore a cornerstone in the management of these patients, and growing evidence shows that implementation of a multidisciplinary team may improve patients’ outcomes. Our aim was to evaluate the clinical and microbiological impact of the application of a multidisciplinary team on critically ill patients.

**Methods:**

Pre-post study enrolling critically ill patients with Gram negative bloodstream infection in intensive care unit. In the pre-intervention phase (from January until December 2018) patients were managed with infectious disease consultation on demand, in the post-intervention phase (from January until December 2022) patients were managed with a daily evaluation by a multidisciplinary team composed of intensivist, infectious disease physician, clinical pharmacologist and microbiologist.

**Results:**

Overall, 135 patients were enrolled during the study period, of them 67 (49.6%) in the pre-intervention phase and 68 (50.4%) in the post-intervention phase. Median age was 67 (58–75) years, sex male was 31.9%. Septic shock, the need for continuous renal replacement therapy and mechanical ventilation at BSI onset were similar in both groups, no difference of multidrug-resistant organisms (MDRO) prevalence was observed. In the post-phase, empirical administration of carbapenems decreased significantly (40.3% vs. 62.7%, *p* = 0.02) with an increase of appropriate empirical therapy (86.9% vs. 55.2%, *p* < 0.001) and a decrease of overall antibiotic treatment (12 vs. 16 days, *p* < 0.001). Despite no differences in delta SOFA and all-cause 30-day mortality, a significant decrease in microbiological failure (10.3% vs. 29.9%, *p* = 0.005) and a new-onset 30-day MDRO colonization (8.3% vs. 36.6%, *p* < 0.001) in the post-phase was reported. At multivariable analysis adjusted for main covariates, the institution of a multidisciplinary management team (MMT) was found to be protective both for new MDRO colonization [OR 0.17, 95%CI(0.05–0.67)] and microbiological failure [OR 0.37, 95%CI (0.14–0.98)].

**Conclusions:**

The institution of a MMT allowed for an optimization of antimicrobial treatments, reflecting to a significant decrease in new MDRO colonization and microbiological failure among critically ill patients.

**Supplementary Information:**

The online version contains supplementary material available at 10.1186/s13613-024-01271-9.

## Background

Bloodstream infections (BSIs) by Gram-negative pathogens play a major role in ICU patients, both in terms of prevalence and severity [1, 2], with reported mortality rates as high as 40% [3]. The choice of an appropriate antibiotic treatment in ICU patients affected by Gram-negative BSIs may be challenging because of the widespread prevalence of multidrug-resistant organisms (MDRO) with high levels of difficult-to-treat resistance (DTR) patterns. More specifically, a recent Italian nationwide study reported extremely high mortality rates if a MDRO is involved, reaching 43%, compared to multi-susceptible Gram-negative BSI [4]. Antimicrobial stewardship has proven to be effective in preventing the rising of antimicrobial resistance, with promising results in ICU patients [5]. It is generally defined as a coordinated interventions designed to improve and measure the appropriate use of antimicrobials by promoting the selection of the optimal antimicrobial drug regimen, dose, duration of therapy, and route of administration [6]. This issue is even more challenging in critically ill patients, where the occurrence of sepsis-related pathophysiological alterations like increased volume of distribution, augmented renal clearance, need for continuous renal replacement therapy (CRRT), have a strong impact on the pharmacokinetic/pharmacodynamic (PK/PD) behaviour of antimicrobials, increasing the risks of under exposure, mainly for beta lactams (BL) [7, 8]. A growing body of evidence shows that early appropriate antibiotic therapy is a cornerstone in the management of septic ICU patients, being associated with a significant decrease in mortality rate [9]. Accordingly, the most recent Surviving Sepsis Campaign guidelines recommend prompt implementation of targeted antibiotic therapy optimized according to pharmacokinetic/pharmacodynamic (PK/PD) principles [10].

In this challenging scenario, a multidisciplinary team composed by the intensivist, the infectious disease (ID) consultant, the clinical pharmacologist and microbiologist provides the best integrated approach for the management of ICU patients and could improve both clinical outcome and antimicrobial overall consumptions, as previously reported [11, 12]. The aim of this study is to assess the impact of a multidisciplinary management team (MMT) on the clinical outcome of ICU patients affected by Gram-negative BSIs.

## Methods

### Study design, setting and participants

This is a pre-post explorative study enrolling critically ill patients with Gram-negative BSIs admitted to general and post-transplant ICUs of the IRCCS S. Orsola-Malpighi hospital, a 1420-bed tertiary teaching hospital located in northern Italy. All consecutive adult (≥ 18 years) ICU admitted patients with a documented Gram-negative BSI were managed according to MMT advice from 1st January 2022 to 31st December 2022. During such study period (namely post-intervention phase) patients were prospectively enrolled and compared to an historical cohort of critically septic patients with Gram-negative BSIs admitted in the same ICUs between 1st January 2018 to 31st December 2018 and treated according to a standard management. A MDRO screening by rectal swab at hospital and ICU admission, as well as weekly during hospitalization, was available in both phases. Similarly, an infection control program and an on demand ID consultant service were active in both phases. Main novel Beta-lactams (i.e. ceftazidime/avibcatam, ceftolozane/tazobactam) were available in both phases.

Documented Gram-negative BSIs were defined as the isolation of a Gram-negative pathogen from at least one blood culture, as previously defined [13]. Only patients with index blood cultures collected during ICU stay or in emergency department on the same day of ICU admission were included. Patients who died within 48 h from index BSI were excluded.

The study was conducted according to the guidelines of the Declaration of Helsinki and approved by the local ethical committee [No. EM 232–2022_308/2021/Oss/AOUBo on 16 March 2022]. Informed signed consent was obtained from all enrolled patients.

### Variables and definitions

Clinical charts and hospital electronic records were used as data sources. Pseudo-anonymous data were retrospectively (pre-intervention) and prospectively (post-intervention) collected using a standard case report form (CRF).

Demographics data (age, sex, and comorbidities), as well as the date of hospital and ICU admission and discharge were collected. SOFA score was calculated at baseline, at 48 h and at 7 days from index BSI. Type of BSI and resistance profile of the pathogen, as well as empirical and targeted treatment were also assessed. Data concerning the attainment of optimal PK/PD targets for antibiotics administered were retrieved. Microbiological failure was defined as breakthrough BSI, recurrent BSI, or persistent BSI during the follow-up period. Persistent BSI was defined as follow-up blood cultures positive for the same pathogen retrieved from index cultures, breakthrough was defined as new positive blood cultures for the same pathogen retrieved from index cultures after negativization. Recurrent BSI was defined as positive blood cultures for the same pathogen retrieved from index cultures after treatment discontinuation. MDRO colonization was defined as the detection of a pathogen with acquired non-susceptibility to at least one agent in three or more antimicrobial categories [14]. A difficult-to-treat resistance (DTR) pathogen was defined as a phenotypic resistance to all first-line agents, following definitions of Kadri et al. [15]. Appropriate antibiotic treatment was defined as a drug with preserved in vitro full susceptibility (i.e. an antibiotic with intermediate MIC was defined as inappropriate) and with favourable PK/PD parameters according to the infection site. Antibiotic appropriateness was mainly evaluated for empirical treatments. Delta 48-hours SOFA was considered as the difference between SOFA score calculated at 48 h and baseline. Similarly, delta 7-days SOFA was considered as the difference between SOFA score calculated at 7 days and baseline. Finally, 30-day mortality and both length of hospital and ICU stay were collected. Patients who died within 24 h from BSI onset were excluded.

### Pre-intervention phase (standard management)

The standard management applied in the pre-intervention phase consisted in the on demand consultation requested by intensivists and carried out only by an ID specialist. Active bedside ID consultations were available during weekends in both periods. As for internal protocol, principles of empirical antibiotic therapy in patients with suspected sepsis were available, selected beta-lactams were usually administered by continuous infusion (CI), although therapeutic drug monitoring (TDM) was rarely performed and no expert interpretation of TDM results provided by Clinical Pharmacologist was available [16].

### Post-intervention phase (intensive multidisciplinary management)

The implementation of the MMT started in late 2019 but became fully operational after mid-2021, when the COVID-19 pandemic slowed down. The MMT consists in the innovative implementation of a multidisciplinary taskforce including the ICU physician, the ID consultant, and the Clinical Pharmacologist which provides a synchronized and coordinated management in all admitted ICU patients, with a specific focus in those with suspected or documented infections, always supported by remote by microbiologist. The members of the multidisciplinary team were always the same physicians, considering that a dedicated ID consultant and a dedicated clinical pharmacologist for the general and post-transplant ICUs provided continuity in the management of critical septic patients, whereas the different ICU physicians belonged to the same team, thus ensuring the implementation of a consistent management for septic cases. If needed, further evaluations of infected patients were performed during the day. Specifically, MMT exhibits a dual role, namely the optimization of antibiotic prescription, dosing, and duration in septic patients, and the improvement in application of infection control measures. The main features of the intensive multidisciplinary management are: daily bedside meeting from Monday-to-Friday in order to allow a daily assessment and reassessment of clinical conditions of patients; prompt update of microbiological culture results at least twice daily through a proactive consultation with microbiologists of preliminary results from blood cultures and other relevant specimens, in the morning and in the afternoon; implementation of a real-time TDM-guided expert clinical pharmacological advice (ECPA) program in the first 72 h in each ICU patient on antibiotic treatment, aimed to provide a tailored antibiotic therapy according to PK/PD principles [16]. Subsequent TDM-guided ECPA reassessments were performed every 48–72 h for evaluating the attainment of optimal PK/PD targets after eventual dosing adjustments or according to variations in pathophysiological conditions. The turnaround time (TAT) for providing a personalized ECPA, defined as the timeframe elapsed between the delivery of TDM blood sample to the laboratory and the publication of the final TDM-guided ECPA in the hospital intranet system, was < 8 h, as previously detailed [16]. Specifically, beta-lactams selected as empirical or targeted therapy are administered by CI, and a steady-state concentration (C_ss_)/MIC ratio > 4 is defined as the best PK/PD target both maximizing clinical/microbiological outcome and minimizing resistance selection [17–19]. In regard to MIC estimation, it was considered the EUCAST clinical breakpoint of the isolated pathogens in blood cultures for the selected agents up to the availability of MIC values (usually 24 h after the identification of the pathogen in blood cultures), and the actual MIC value of the clinical isolate as soon as available.

### Outcomes

The primary endpoints were: (i) rates of inappropriate empirical therapy, (ii) microbiological failure, (iii) incidence of new MDRO colonization during the follow-up period in both phases. Secondary endpoints were: improvement in delta-48 h SOFA, delta 7-day SOFA, clinical cure at 7 days (defined as signs and symptoms resolution along with biochemical parameters reduction/normalization within 7 days from BSI onset), total and median defined daily dose (DDD)/patient for main antibiotics, and 30-day mortality rates in the two groups.

### Statistical analysis

Categorical variables were expressed as absolute numbers and their relative frequencies. Continuous variables were expressed as mean ± standard deviation (SD) if normally distributed, or as median and interquartile range (IQR) if non-normally distributed. At univariable analysis, categorical variables were compared using Pearson chi‐square or Fisher’s exact test where appropriate. Continuous variables were compared using Student’s t or Mann‐Whitney U test according to their distribution. A logistic regression analysis was performed in order to identify factors associated with microbiological failure and MDRO carriage including clinically relevant or statistically significant variables identified at univariate analysis.

## Results

A total of 629 and 685 patients were admitted to our ICU during the two study periods (2018 and 2022), respectively. Of those, 67 in the pre-phase and 68 in the post-phase had a documented Gram-negative BSI and were therefore enrolled in the present study (see Table 1 and Supplementary Figure E1). No significant differences in terms of median age (67 vs. 64 years), male proportion (34.3% vs. 29.4%), and median Charlson comorbidity index (5 vs. 5) were reported between pre- and post-intervention phase. In both cohorts, no significant differences emerged in regard to proportion of septic shock (61.2% vs. 69.1%), and requirement for CRRT (43.3% vs. 35.3%) and mechanical ventilation (64.2% vs. 72.1%) at BSI onset. A significantly higher median SOFA score at baseline was found in patients included in the post-intervention phase (10 vs. 8 points; *p* = 0.02). The majority of BSI were classified as hospital-acquired (105, 77.8%), with a median timing of occurrence of 2 (0–13) days from ICU admission. Patients in the post-phase had an increased probability of hospital-acquired BSI (85.3% vs. 70.1%, *p* = 0.05). Overall, secondary BSI accounted for 73.7% of cases, mainly from an intra-abdominal and lower respiratory tract source (45.9% and 34.7%, respectively). In 122 (91%) patients, blood cultures yielded *Enterobacterales*, followed by *Pseudomonas* spp. (8, 6%) and *Acinetobacter* spp. (3, 2.2%) with similar distribution of MDRO in both groups. In a quarter of cases (33, 24.4%), pathogens were classified as DTR (Table 2). Overall, an empirical treatment based on beta-lactam/beta-lactamase inhibitor (BL/BLI) or a carbapenem was administered in 42 (31.3%) and 66 (49.3%) of patients, respectively, with an overall rate of appropriate empirical therapy of 71.1%. Empirical treatment was suggested by the MMT in 58 out of 68 cases (85.3%), while in the remaining 10 cases was promptly revised within 12 h. The median duration of appropriate therapy was 14 [11–17] days. Eighty-two (61.7%) patients reached clinical cure at seven days, with median delta 7-days SOFA of 2 [1–5] from baseline. All-cause 30-day mortality was 35.6%, in 29 (31.9%) patients a new colonization by a MDRO was detected by surveillance cultures. Finally, a fifth of patients (27, 20.0%) had a microbiological failure.A comparison between pre- and post-intervention phases is shown in Table 1. In the post-phase, the empirical administration of carbapenems decreased significantly (40.3% vs. 62.7%, *p* = 0.02) along with an increase of appropriate empirical therapy (86.9% vs. 55.2%, *p* < 0.001). Conversely, empirical prescription of BL/BLIs significantly increased (19.4% vs. 43.3%). Overall, a significant decrease in median antibiotic treatment duration (12 vs. 16 days, *p* < 0.001) was reported. Similarly, DDD/patient for meropenem showed a significant decrease. Considering the management of the infection, follow-up blood cultures were performed in 97.1% of patients in the post-phase, and in 95.6% of such cases a microbiological clearance was documented. In the post-intervention period all patients reached the PK/PD target attainment both at 48 h and 7 days of treatment.

**Table 1:** Comparison between pre- and post-intervention phase among critically ill patients with gram negative bloodstream infection

	Pre-phase (N = 67, 49.6%)	Post-phase (N = 68, 50.4%)	Overall (N = 135)	p-value
**Demographic data**				
Age (years), median (IQR)	67 (55–75)	64 (59–75)	67 (58–75)	0.85
Sex, male	23 (34.3)	20 (29.4)	43 (31.9)	0.58
**Baseline characteristics**				
MDR colonization	19 (28.4)	17 (25.0)	36 (26.7)	0.70
Immunosuppression	13 (19.4)	20 (29.4)	33 (24.4)	0.23
Charlson comorbidity index, median (IQR)	5 (3–7)	5 (4–7)	5 (3–7)	0.19
**Infection characteristics**				
Septic shock	41 (61.2)	47 (69.1)	88 (65.2)	0.22
CRRT	29 (43.3)	24 (35.3)	53 (39.3)	0.38
MV	43 (64.2)	49 (72.1)	92 (68.1)	0.36
Baseline SOFA score	8 (6–9)	10 (6–14)	8 (6–11)	0.02
Pitt score	2 (0–6)	2 (0–3)	2 (0–4)	0.06
**BSI characteristics**				
Community-acquired	9 (13.4)	7 (10.3)	16 (11.9)	0.05
Healthcare-related	11 (16.4)	3 (4.4)	14 (10.4)	
Hospital-acquired	47 (70.1)	58 (85.3)	105 (77.8)	
Primary	10 (14.9)	13 (19.1)	23 (17.0)	0.32
Secondary	48 (73.8)	50 (73.5)	98 (72.6)	0.03
Intra-abdominal	27 (56.3)	18 (36)	45 (45.9)	
LRTI	11 (22.9)	23 (46.0)	34 (34.7)	
UTI	8 (16.7)	9 (18.0)	17 (17.3)	
Other	2 (4.2)	0 (0)	2 (2.0)	
Device-related	9 (13.8)	5 (7.4)	14 (10.4)	
ICU admission to BSI onset (days) (median, IQR)	4 (1–14)	2 (0–13)	2 (0–13)	0.26
**Management of BSI**				
FUBC	40 (59.7)	66 (97.1)	106 (78.5)	< 0.001
Negative FUBC	25 (62.5)	65 (95.6)	90 (83.3)	< 0.001
SC performed	9 (1.2)	5 (1.4)	14 (10.3)	0.11
SC not performed	13 (19.4)	7 (10.3)	20 (14.8)	
SC not applicable	29 (43.3)	41 (60.3)	70 (51.9)	
**Antibiotic management**				
Empirical cephalosporin	4 (6.0)	8 (11.9)	12 (9.0)	0.37
Empirical BL/BLI	13 (19.4)	29 (43.3)	42 (31.3)	0.005
Empirical carbapenem	42 (62.7)	27 (40.3)	69 (51.5)	0.02
Appropriate empirical therapy	37 (55.2)	59 (86.9)	96 (71.1)	< 0.001
Targeted cephalosporin	5 (7.6)	16 (23.5)	21 (15.6)	0.001
Targeted BL/BLI	9 (13.6)	18 (26.5)	27 (20.1)	0.08
Targeted novel BL/BLI	14 (20.9)	10 (14.7)	28 (17.8)	0.23
Targeted carbapenem	37 (56.1)	29 (42.6)	66 (49.3)	0.17
Targeted escalation	3 (4.5)	2 (2.9)	5 (3.7)	0.98
Targeted de-escalation	8 (11.9)	14 (20.6)	22 (16.3)	0.56
Days of appropriate therapy (median, IQR)	16 (12–20)	12 (9–15)	14 (11–17)	< 0.001
C_ss_/MIC ratio > 4 at 48 h from B-lactam start	-	68 (100)		
C_ss_/MIC ratio > 4 at 7 days from B-lactam start	-	68 (100)		
Dosing reduction after first TDM assessment	-	43 (63.2)		
Overall piperacillin/tazobactam DDD/patient	105	168	273	0.002
Median piperacillin/tazobactam DDD/patient	2 (2–12)	3 (2–6)	3 (2–7)	0.78
Overall meropenem DDD/patient	585	232	814	< 0.001
Median meropenem DDD/patient	15 (11–19)	7 (3–10)	11 (7–17)	< 0.001
**Outcomes**				
SOFA 48 h	7 (5–9)	8 (5–14)	7 (5–10)	0.15
Delta SOFA 0-48 h	1 (-1-2)	1 (0–2)	1 (0–2)	0.57
SOFA score 7 days	4 (1–7)	6 (3–10)	5 (2–8)	0.002
Delta SOFA 0-7days	2 (0–4)	3 (1–6)	2 (1–5)	0.26
Clinical cure	31 (47.7)	51 (75.0)	82 (61.7)	0.001
Microbiological failure	20 (29.9)	7 (10.3)	27 (20.0)	0.005
Persistent BSI	11 (16.4)	3 (4.4)	14 (10.4)	0.03
Breakthrough BSI	10 (14.9)	4 (5.9)	14 (10.4)	0.09
Recurrent BSI	12 (17.9)	5 (7.4)	17 (12.6)	0.07
MDRO colonization 30-day	23 (53.5)	6 (12.5)	29 (31.9)	< 0.001
New MDRO colonization 30-day	15 (36.6)	4 (8.3)	19 (21.3)	0.002
MDRO colonization 90-day	13 (36.1)	4 (9.8)	17 (22.1)	0.007
C. difficile infection 30-day	1 (2.6)	2 (4.2)	3 (3.5)	1
All-cause 30-day mortality	24 (35.8)	24 (35.3)	48 (35.6)	1
All-cause 90-day mortality	7 (17.1)	3 (6.8)	10 (11.8)	0.19
Length of hospital stay	42 (24–64)	43 (27–64)	43 (26–64)	0.46
Length of ICU stay	21 (7–36)	18 (6–34)	18 (7–35)	0.41

All values given are n (%) unless otherwise stated

*Abbreviations* IQR interquartile range, MDRO multidrug-resistant organism, SOFA sequential organ failure assessment, CRRT continuous renal replacement therapy, Css steady state concentration, MIC minimum inhibitory concentration, MV mechanical ventilation, BSI bloodstream infection, LRTI lower respiratory tract infection, UTI urinary tract infection, FUBC follow-up blood cultures, SC source control, BL/BLI beta-lactam/beta-lactam inhibitor, ICU intensive care unit

**Table 2:** Main pathogens yielded from blood cultures and relative patterns of resistance between periods

	Pre-phase (N = 67, 49.6%)	Post-phase (N = 68, 50.4%)	Overall (N = 135)	p-value
**Etiology**				0.06
Enterobacteriaceae	63 (95.5)	59 (86.8)	122 (91)	0.15
*Klebsiella* spp.	39 (61.9)	27 (45.8)	66 (54.1)	
*E. coli*	12 (19.0)	16 (27.1)	28 (23.0)	
*Enterobacter* spp.	8 (12.7)	6 (10.2)	14 (11.5)	
*Proteus* spp.	3 (4.8)	2 (3.4)	5 (4.1)	
*Serratia* spp.	0 (0)	3 (5.1)	3 (2.5)	
*Pseudomonas* spp.	1 (1.5)	7 (10.3)	8 (6.0)	
*Acinetobacter* spp.	1 (1.5)	2 (2.9)	3 (2.2)	
**Pattern of resistance**				
Wild-type	26 (40.0)	32 (47.1)	58 (43.6)	0.49
ESBL-E	18 (27.7)	18 (26.5)	36 (27.1)	1
Carbapenem-resistant	18 (27.7)	11 (16.2)	29 (21.8)	0.14
KPC	17 (25.4)	8 (11.8)	25 (18.5)	0.002
DTR	19 (28.4)	15 (22.1)	34 (25.2)	0.32

All values given are n (%) unless otherwise stated

*Abbreviations* ESBL-E extended spectrum beta-lactamase Enterobacterales, DTR difficult-to-treat resistance

No differences in delta 48-hours SOFA and delta 7-days SOFA were reported in both groups. Similarly, rates of all-cause 30-day and 90-day mortality, as well as length of ICU stay were comparable. Clinical cure was increased in the post-phase (75% vs. 47.7%, *p* = 0.001). A significant decrease of microbiological failure (10.3% vs. 29.9%, *p* = 0.005) and a new-onset 30-day MDRO colonization (8.3% vs. 36.6%, *p* < 0.001) in the post-phase was reported.

At multivariable analysis adjusted for main covariates, MMT was found to be protective both for new MDRO colonization [OR 0.17, 95%CI (0.05–0.67)] and microbiological failure [OR 0.37, 95%CI (0.14–0.98)] (Tables 3 and 4 and Tables E1 and E2, *online supplement*).

**Table 3:** Multivariable analysis for new MDRO colonization adjusted for age, sex, CCI, MMT, appropriate empirical therapy and clinical cure

Variable	OR	95%CI	p
MMT	0.17	(0.05–0.67)	0.010
Clinical cure	0.13	(0.03–0.58)	0.007

*Abbreviations* CCI Charlson comorbidity index, MMT multidisciplinary management team, OR odds ratio, CI confidence interval

**Table 4:** Multivariable analysis for microbiological failure adjusted for age, sex, CCI, MMT, DTR and appropriate empirical therapy

Variable	OR	95%CI	p
MMT	0.37	(0.14–0.98)	0.042
DTR	2.71	(1.03–7.15)	0.043

*Abbreviations* CCI Charlson comorbidity index, MMT multidisciplinary management team, DTR difficult-to-treat resistance, OR odds ratio, CI confidence interval

## Discussion

In our pre-post explorative study the implementation of a MMT allowed to significantly increase the rate of appropriate empirical therapy, decrease empiric carbapenems administration, and reduced overall antibiotic treatment duration in critically septic patients affected by Gram-negative BSIs. Notably, this resulted in a significant reduction in microbiological failure and new MDRO colonization, thus potentially supporting the remarkable role of an MMT as an effective tool for antimicrobial stewardship programs for ICU setting.

Data from different randomized clinical trials suggest that approximately 30–40% patients admitted in ICU with sepsis or septic shock are bacteraemic [2]. In the last few decades, a significant shift from Gram-positive to Gram-negative BSI prevalence in ICU has been recorded, together with an ominous increase of MDRO circulation [20]. Accordingly, we decided to include only patients with Gram-negative BSI. It has been calculated that the rate of Gram-negative ICU-acquired BSI is approximately of 6.9/1000 admissions and 0.97–1.1/1000 patient-days [21, 22]. Such infections, especially if a MDRO is involved, are burdened by poor outcomes, reaching ICU mortality rates from 20% up to 50% [22, 23], thus supporting the importance of implementing novel tools for improving clinical and microbiological outcome in critically ill patients affected by Gram-negative BSIs.

Several studies have already stressed the impact of a prompt and appropriate empirical therapy on short-term survival of critically ill patients [10, 24]. Our intervention allowed to significantly increase the rate of an appropriate empirical therapy, from less than 60% in the pre-intervention phase, to almost 90% in the post-intervention phase. The low rate of appropriate empirical treatment in the pre-intervention phase was mainly due to a consistent prevalence of difficult-to-treat pathogens amongst isolates. Of interest, such an increase in antibiotic appropriateness was obtained despite of a significant decrease in empirical carbapenem prescription (62.7% vs. 40.3%). Novel BL/BLIs (i.e., ceftolozane-tazobactam and ceftazidime-avibactam) were only reserved for the management of carbapenem-resistant pathogens and not as carbapenem-sparing strategy according to our local and national epidemiology. Consequently, this should not have contributed to the lower carbapenem use in the post-period. As previously described, carbapenem exposure is one of the main drivers for subsequent carbapenem-resistant microorganisms acquisition [25]. Therefore, optimization of empirical treatment could be one of the reasons why we observed a significant decrease of new MDRO carriage.

Another relevant issue is the duration of antibiotic therapy, that was significantly lower in the post-intervention phase. In contrast to what performed in the pre-intervention period, the adoption of a full maintenance beta-lactam dosing also in cases exhibiting sepsis-related acute kidney injury coupled with the implementation of a real-time TDM-guided ECPA strategy allowed the early attainment of optimal beta-lactam PK/PD targets in all patients managed according to the MMT. Surviving Sepsis Campaign guidelines strongly recommended antimicrobial dosing optimization according to PK/PD principles and specific drug properties in septic ICU patients [10]. Notably, several pre-clinical and clinical evidence reported that the attainment of aggressive PK/PD targets with beta-lactams (at least a 100%T_> 4xMIC_) may allow to maximize clinical outcome and suppress resistance emergence. In this scenario, the achievement of optimal beta-lactam PK/PD targets at both 48 h and 7-day after BSI onset in our MMT patients may have contributed to improve microbiological outcomes and therefore reduce days of antibiotic therapy. Presumably, the optimization of antibiotic therapy may have influenced the substantial decrease in persistent, breakthrough and recurrent BSI. Indeed, an antibiotic therapy tailored on the specific patient characteristics along with a shortening of the duration of antimicrobial exposure are the cornerstones of antimicrobial stewardship programs [26, 27]. Although the lower proportion of secondary BSIs associated with an intra-abdominal source in the post-intervention phase could partially explain the significant reduction observed in terms of duration of antibiotic therapy, it is noteworthy that the achievement of an effective source control represents a cornerstone in the management of Gram-negative BSI, and that this issue was strictly associated with FUBC performance [28]. Considering the higher proportion of patients underwent to FUBCs coupled with a lower proportion of cases in which source control was not performed reported in the post-intervention phase, it could not be ruled out that these factors could have played a major role in shortening antibiotic treatment duration. Although performing FUBCs in Gram-negative infections still remains a debated issue, recent meta-analyses found that this procedure was associated with significant reduction in mortality rate [28, 29], thus FUBCs may be recommended for the management of critical patients affected by Gram-negative BSIs. However, for optimizing health resources, a proposed risk score could be applied for promptly identifying cases at high-risk for persistent BSIs who may benefit from FUBCs execution [28].

One of the most important and promising result we observed is the significant reduction of new MDRO carriage in ICU patients within 30 days from BSI index. Different studies highlighted the dramatic impact that MDRO colonization could have in critically ill patients [30, 31]. Thus, ICU patients with a MDRO infection have an increased risk of short-term mortality compared with non-MDRO patients or uninfected [32, 33]. Therefore, MDRO acquisition or transmission should be avoided. We believe that the institution of the MMT has led to such a result, operating at two different levels: optimizing antibiotic prescription, dose, and duration in infected patients, as well as allowing a better observation of in-hospital infection control protocols, although adherence was not formally assessed in our analysis.

Despite an increase in empirical appropriateness, we did not observe a significant improvement in delta 48-hours and 7-day SOFA nor 30-day mortality. Although appropriate empirical therapy generally plays a key role in infected critically ill patients, some studies specifically focused on Gram-negative bacteraemia, such as *E. coli* and *K. pneumoniae*, did not find an association between appropriate empirical therapy and lower short-term mortality [34–36]. This is not surprising, considering that a wide-spectrum therapy works as well as a narrowed one, if we consider only the short-time clinical improvement as primary outcome. Furthermore, the significantly higher severity in terms of SOFA score at BSI onset retrieved in patients enrolled in the post-intervention phase could also partially explain the absence of a significant impact of MMT on mortality. However, prescribing an antibiotic therapy the physician is asked to maintain a responsible behaviour, always considering the potential ecological impact related to antibiotics overexposure. Likewise, 30-day mortality in ICU patients could be influenced by more specific patient-related conditions than appropriate empirical therapy, and therefore not representing an optimal impact parameter. Noteworthy, although the relative low sample size, the subgroup of patients in which the MTT had a remarkable impact are immunosuppressed patients, considering a trend toward lower mortality rates (20.0% vs. 30.8%, *p* = 0.68), both lower rates of microbiological failure (5% vs. 38.5%, *p* = 0.03) and new MDRO colonization (6.3% vs. 33.3%, *p* = 0.12).

Overall, the significant lower risk of microbiological failure and the reduction in new MDRO colonization reported in our study may support the importance of implementing a coordinated and synchronized MMT as a tool for antimicrobial stewardship programs in ICU, in order to both minimize microbiological selective pressure and ecologic/economic costs related to the use of novel beta-lactams. It is noteworthy that the presence of the ICU physician in the multidisciplinary taskforce played a crucial role in the successful implementation of our antimicrobial stewardship program, considering that advices provided by the ID consultant and the Clinical Pharmacologist were shared and applied in all cases.

Limitations of our study have to be addressed. The relatively small sample size and an overall more severe baseline conditions at infection onset probably did not allow us to demonstrate an impact of the MMT implementation both in terms of SOFA score reduction and 30-day mortality. However, a trend toward a decrease in delta 48-hours SOFA score was observed and rates of clinical cure were significantly higher in the post-intervention phase. The monocentric design of our study could prevent the extensive generalization of our findings in different ICU settings. Furthermore, we considered two non-consecutive years, which may have contributed to some biases, but we preferred to exclude COVID-19 patients, in order to reduce the number of possible confounders. However, fast microbiology, that could significantly affect treatment appropriateness, was only implemented for a restricted number of patients with pneumonia, and thus excluded from the study population, and multiplex PCR for bloodstream infection was not available in both phases. Secondly, infection control protocols and overall in-hospital rates of antimicrobial resistance were unchanged during both phases. Rates of CPE colonized patients in the overall hospital setting were similar in both phases (4.1% in 2018 vs. 3.0% in 2022), consistent with baseline rates of MDRO colonized patients at ICU admission (28.4% vs. 25.0%) in our study population. However, we observed a decrease in colonization rates in the overall ICU population, from 10.1% in 2018 to 3.7% in 2022. The latter issue may suggest the potential role of the MTT in the decrease of new MDRO colonization rates observed in the post-phase. In addition, the selection of ICU patients with Gram-negative BSI occurrence in 2018 represents a reliable comparative arm considering that both ICU physicians and ID consultants remained unchanged during study period, thus ensuring consistent clinical expertise and care, and novel agents for the management of MDRO Gram-negative infections were available in both periods. Finally, the significant increase in hospital acquired BSIs in the post-intervention phase may have played a role in shifting the prevalence of some pathogens, such as *P. aeruginosa*, although the impact of other concomitant factors cannot be ruled out.

## Conclusion

In conclusion, our study highlights the relevance of implementing an MMT provided by a multidisciplinary taskforce composed by intensive care physicians, ID consultant, and MD clinical pharmacology specialists in the ICU setting, potentially impacting on the appropriateness of empirical antibiotic therapy, optimization of antimicrobial prescription and MDRO carriage acquisition, from the perspective of an antimicrobial stewardship program.

### Electronic supplementary material

Below is the link to the electronic supplementary material.

Supplementary Material 1: Study flow chart

Supplementary Material 2: Supplementary tables E1 and E2

## Data Availability

The datasets used and/or analysed during the current study are available from the corresponding author on reasonable request.

## Abbreviations

BL: beta-lactam
BLI: beta-lactamase inhibitor
BSI: blood stream infection
CI: confidence interval
CRF: case report form
CRRT: continuous renal replacement therapy
C_ss_: steady state concentration
DDD: defined daily dose
DTR: difficult-to-treat resistance
ECPA: expert clinical pharmacological advice
ICU: intensive care unit
ID: infectious disease
IQR: inter-quartile range
MDRO: multidrug-resistant organism
MIC: minimum inhibitory concentration
MMT: multidisciplinary management team
PD: pharmacodynamic
PK: pharmacokinetic
SD: standard deviation
SOFA: Sequential Organ Failure Assessment
TDM: therapeutic drug monitoring
