# Supplementary figures and images for: Impact of a multidisciplinary management team on clinical outcome in ICU patients affected by Gram-negative bloodstream infections: a pre-post quasi-experimental study
Source: Ann Intensive Care. 2024 Mar 6;14:36. doi: 10.1186/s13613-024-01271-9 (PMC10917714; doi:10.1186/s13613-024-01271-9)

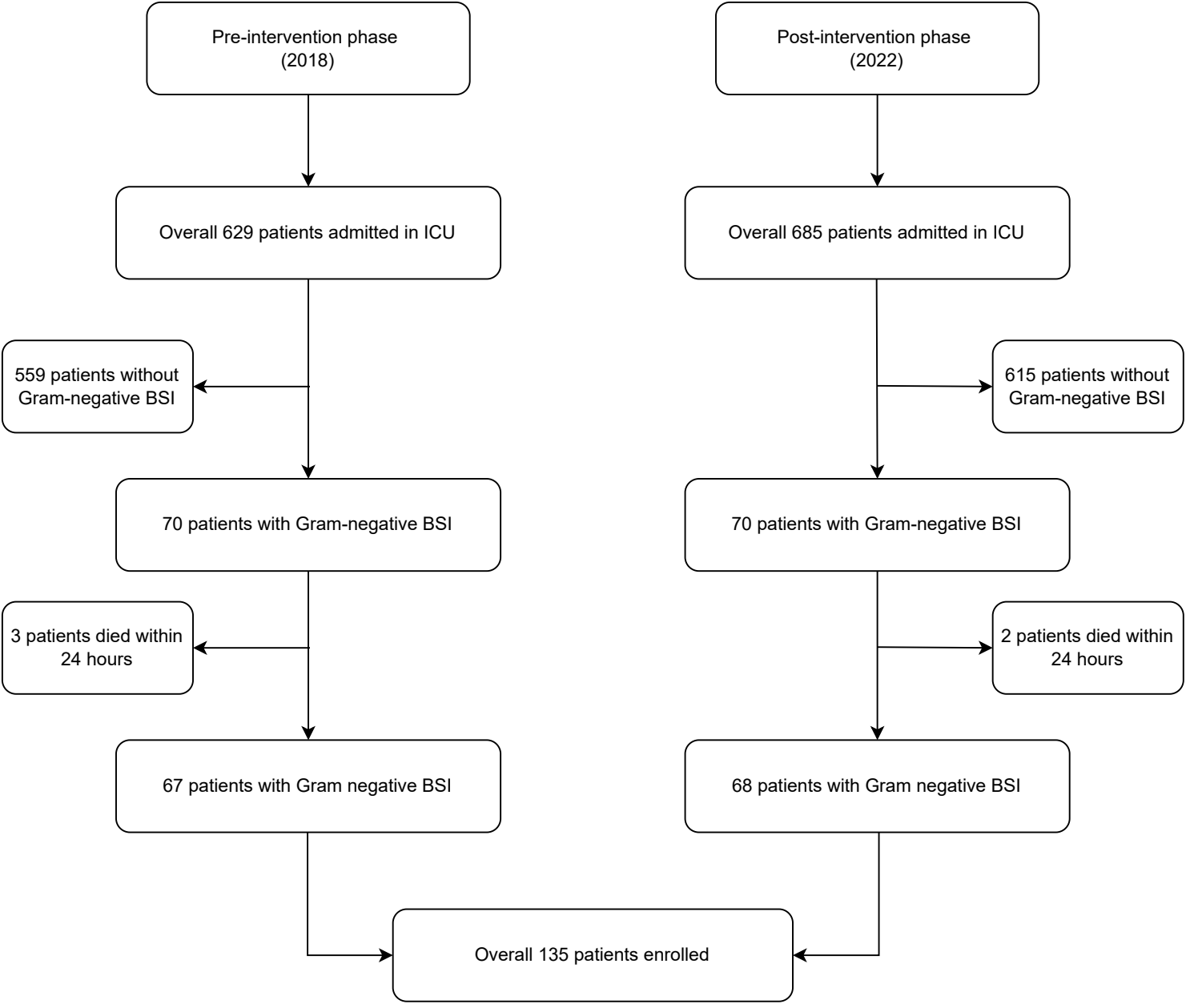

Supplement: Supplementary file 1 — Supplementary Material 1: Study flow chart [file 13613_2024_1271_MOESM1_ESM.pdf]
