# Supplementary material for: Impact of a multidisciplinary management team on clinical outcome in ICU patients affected by Gram-negative bloodstream infections: a pre-post quasi-experimental study
Source: Ann Intensive Care. 2024 Mar 6;14:36. doi: 10.1186/s13613-024-01271-9 (PMC10917714; doi:10.1186/s13613-024-01271-9)
Supplement: Supplementary file 2 — Supplementary Material 2: Supplementary tables E1 and E2 [file 13613_2024_1271_MOESM2_ESM.docx]

**Suppl. Table 1**. Unadjusted regression logistic analysis for new MDRO colonization.

| **Variable** | **OR** | **95%CI** | **p** |
| --- | --- | --- | --- |
| Age | 0.99 | (0.96-1-03) | 0.688 |
| Sex (male) | 1.37 | (0.44-4.27) | 0.587 |
| CCI | 0.98 | (0.79-1.19) | 0.748 |
| MMT | 0.11 | (0.08-0.15) | 0.002 |
| AET | 0.97 | (0.94-1.01) | 0.589 |
| Clinical cure | 0.08 | (0.02-0.14) | 0.001 |

Abbr.: CCI Charlson comorbidity index, MMT multidisciplinary management team, AET appropriate empirical therapy, OR odds ratio, CI confidence interval.

**Suppl. Table 2**. Unadjusted regression logistic for microbiological failure.

| **Variable** | **OR** | **95%CI** | **p** |
| --- | --- | --- | --- |
| Age | 1.00 | (0.97-1.04) | 0.837 |
| Sex (male) | 1.14 | (0.45-2.86) | 0.782 |
| CCI | 1.07 | (0.90-1.28) | 0.444 |
| AET | 0.84 | (0.66-1.02) | 0.122 |
| MMT | 0.27 | (0.11-0.69) | 0.006 |
| DTR | 3.31 | (1.35-8.12) | 0.009 |

Abbr.: CCI Charlson comorbidity index, AET appropriate empirical therapy, MMT multidisciplinary management team, DTR difficult-to-treat resistance, OR odds ratio, CI confidence interval.
